# Evaluation of cell surface reactive immuno-adjuvant in combination with immunogenic cell death inducing drug for in situ chemo-immunotherapy

**DOI:** 10.1016/j.jconrel.2020.03.029

**Published:** 2020-06-10

**Authors:** Adam A. Walters, Julie Tzu-Wen Wang, Khuloud T. Al-Jamal

**Affiliations:** Institute of Pharmaceutical Science, Faculty of Life Sciences & Medicine, King's College London, Franklin-Wilkins Building, 150 Stamford Street, London SE1 9NH, United Kingdom

**Keywords:** Apoptotic body, Intratumoral, CpG, Apoptosis, Doxorubicin, Cholesterol conjugate, Dendritic cell

## Abstract

Apoptotic cells and cell fragments, especially those produced as a result of immunogenic cell death (ICD), are known to be a potential source of cancer vaccine immunogen. However, due to variation between tumours and between individuals, methods to generate such preparations may require extensive *ex vivo* personalisation. To address this, we have utilised the concept of *in situ* vaccination whereby an ICD inducing drug is injected locally to generate immunogenic apoptotic fragments/cells. These fragments are then adjuvanted by a co-administered cell reactive CpG adjuvant. We first evaluate means of labelling tumour cells with CpG adjuvant, we then go on to demonstrate *in vitro* that labelling is preserved following apoptosis and, furthermore, that the apoptotic body-adjuvant complexes are readily transferred to macrophages. In *in vivo* studies we observe synergistic tumour growth delays and elevated levels of CD4+ and CD8+ cells in tumours receiving adjuvant drug combination. CD4+/CD8+ cells are likewise elevated in the tumour draining lymph node and activated to a greater extent than individual treatments. This study represents the first steps toward the evaluation of rationally formulated drug-adjuvant combinations for *in situ* chemo-immunotherapy.

## Graphical abstract

**Figure:**
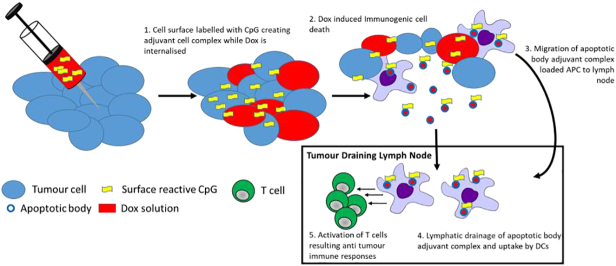

## 1 Introduction

Apoptotic cancer cells are believed to be a potential source of immunogen for cancer vaccination which, in some models, though not all, have been shown to be superior to both necrotic cells and whole cell lysate at eliciting an immune response [[1], [2], [3], [4]]. Pre-clinically, these vaccines are generated by inducing apoptosis in a parental cell line *in vitro* (through radiation or drug treatment) then administering them *in vivo* to mice implanted with homologous tumour. The cell preparation is typically administered as either a whole apoptotic fraction, or in the form of a pulsed dendritic cell vaccine where apoptotic cells serve as an antigen source [5,6]. Approaches such as these have yielded promising results pre-clinically.

When producing apoptotic fraction for use as a vaccine the mechanism by which apoptosis is induced is an important consideration and recently the use of immunogenic cell death (ICD) inducers to initiate apoptosis has been gaining prominence. ICD is a form of apoptosis arising from treatment of cells with certain therapeutics, such as doxorubicin and oxaliplatin or radiation [7]. ICD has been well described in preclinical models but has not been generally observed clinically, which, some have speculated, may be due to the drug doses required to induce ICD being near the maximal tolerated dose [8]. Physiologically ICD is characterised by release of inflammatory mediators, such as ATP and HMGB1, and the translocation of calreticulin to the cell surface [9]. These molecules serve as immunostimulants in the case of ATP and HMGB1, activating inflammatory pathways and TLR4, and, so called ‘eat me’ signals, in the case of calreticulin, serving to increase phagocytosis of the dying cell [10]. More relevantly, the gold standard test for ICD is the prophylactic vaccination against homologous tumours using cells undergoing ICD as the immunogen [10,11]. For this reason, the use of apoptotic fractions produced as a result of ICD have also been proposed as a potential cancer vaccine [12,13]. Indeed, groups have used ICD induced cells as a source of antigen for dendritic cell vaccines [14,15].

Interestingly, for both non-ICD and ICD induced apoptotic cells, groups have worked on improving the potency of these vaccines further through direct conjugation of adjuvants such as TLR9 agonist CpG to the apoptotic cell/fraction surface [13,16]. Such apoptotic cell-adjuvant complexes are immunogenic and have shown protection in various tumour challenge models. ICD induced cell-CpG complexes are especially promising and result in both suppression of tumour growth and potent systemic immunity [13]. However, while the use of apoptotic cells as vaccines has been successful in preclinical models, clinically, in a therapeutic setting, the development of such approaches may require extensive personalisation. For instance, the generation of apoptotic fractions will be dependent on isolating cells from biopsies, *ex vivo* culturing them, treating them with an indeterminate quantity of ICD inducer (depending on tumour sensitivity) and re-administering them to the patient. This will require elaborate processing and quality control which may hinder its eventual translation.

In parallel to the to the rise in interest in ICD, *in situ* vaccination is growing in popularity within the literature. In this process an immuno-adjuvant is injected directly into the tumour to stimulate the immune system locally. The immune responses generated locally will then lead to the establishment of systemic immunity, resulting in the clearance of secondary metastases, and the production of an immunological memory protecting from remission. This is a powerful concept because it is based upon the understanding that the tumour microenvironment is rich in tumour specific immune cells and personal tumour antigen. It therefore focuses on relieving the immunosuppression generated by the tumour or by directly stimulating local immune cell populations. A range of modalities have been tested for this purpose including plant viruses, classical adjuvants, monoclonal antibodies or combinations of the above [17,18]. The use of *in situ* vaccination as opposed to ‘traditional’ cancer vaccination is particularly attractive as it circumvents the requirement for labour intensive ‘personalised’ vaccines such as those based on apoptotic cells.

The combination of *in situ* apoptotic cell-based vaccine where the apoptotic cells are generated *in situ* has yet to be explored. This manuscript explores the potential of such a concept. Based on the intratumoral injection of ICD inducer in combination with a cell labelling immuno-adjuvant (CpG) we expect to generate an ‘*in situ* apoptotic cell-adjuvant complex’. We hypothesise that this approach will feature both the direct cytotoxic effects of the drug on the tumour and improved systemic immuno-stimulation effect due to the presence, and co-localisation of, adjuvant and apoptotic fraction.

## 2 Materials and methods

### 2.1 Materials

CpG (ODN 1668, 5′-tccatgacgttcctgatgct-3′ all bases phosphorothioated) constructs were prepared commercially as described in [19], where required the 3′ terminus was modified with cholesterol residue or amine group (Eurogentec, Belgium). For imaging, the 5′ terminal was modified with Alexa647 or Cy5. Doxorubicin (Dox) was purchased from Apollo Scientific (UK). Ellmans reagent and sulfo succinimidyl 4-(N-maleimidomethyl) cyclohexane-1-carboxylate (sulfo-SMCC) were purchased from Thermo Scientific (UK). Maleimide Alexa488 and Azide Alexa488 was purchased from Invitrogen (UK). Maleimide Quantification Assay kit was purchased from Abcam. Antibodies (anti-mouse CD4-FITC (clone GK1.5), anti-mouse CD8-PE (clone 53–6.7) and anti-mouse CD69-APC (clone H1.2F3)), precision count beads and annexin V staining kit were purchased from Biolegend (UK). Cell culture reagents: Glutamax, penicillin/streptomycin, RPMI medium and FBS were purchased from Gibco (Thermo Fisher Scientific, UK). Amicon ultra 3 k MWCO were purchased from Merck Millipore (Germany). TCEP, cysteine and all other laboratory chemicals were purchased from Sigma (UK).

### 2.2 Cell culture

CT26 murine colon carcinoma cells and J774 macrophage were maintained in RPMI media supplemented with 10% FBS, 50 U/ml penicillin, 50 μg/ml streptomycin and 1% Glutamax. Cells were incubated in a humidified incubator in 5% CO_2_ at 37 °C.

### 2.3 Animals

All animal experiments were conducted in agreement with the existing personal and project licenses granted by the UK Home Office and in accordance with the UKCCCR Guidelines (1998). Female BALB/c mice ages 4–6 weeks were purchased from Envigo (UK).

### 2.4 Assessment of cell surface thiol residues

Cell surface thiol residues were quantified using Ellman's reagent. In brief, Ellman's working reagent was added to CT26 cells which had been titrated from 10^7^ to 1.5^5^ absolute cell number. The reaction was incubated for 15 mins before cells were pelleted and the supernatant was removed. The supernatant OD_412nm_ was read using a Fluorstar plate reader (BMG Labtech). Cysteine was used to establish a standard curve. As further validation of surface thiol residues, CT26 cells were incubated with 10 μg Maleimide-Alexa 488 (Mal-Alexa488). To remove excess unreacted Mal-Alexa 488, cells were washed twice with PBS. Cells were acquired using a FACs Calibur flow cytometer. As a negative control, the reaction was performed with a 100-fold molar excess of cysteine. The reaction was also performed in the presence of reducing agent TCEP and buffer at pH 6.5 as an alternative condition.

For *in vivo* accessibility of surface thiols to Mal-CpG, BALB/c mice were implanted with CT26 tumours, at 7 days post implantation Mal-Alexa488 was injected intratumourally (i.t.) at a dose of 10 μg/tumour. Azide Alexa 488 was used at a corresponding dose as a negative control. At 24 h post injection, tumours were extracted, and cells were extracted by physical dissociation by maceration of tissue through a cell strainer. The cells were next acquired on a FACs Calibur flow cytometer. Fluorescence of cells extracted from the Mal Alexa 488 were compared to those extracted from Azide Alexa 448 using Flowjo software.

### 2.5 Preparation of CpG constructs

CpG-Chol and Alexa647/Cy5-CpG-Chol was used as obtained with no further modification. To modify CpG with Maleimide, aminated CpG purchased from Eurogentec was reacted with sulfo SMCC. In brief aminated CpG in phosphate buffer was mixed with sulfo SMCC at a 100-fold molar excess. The reaction was allowed to proceed for one hour at room temperature. The construct was purified and washed 3 times using Amicon Ultra 3 k MWCO spin columns. To confirm the reaction had been successful maleimide residues were quantified using a maleimide detection kit in accordance with manufacturer's protocol (Abcam). In brief, MalemGreen indicator was added to test samples to a obtain a final concentration of 1×. The reaction was incubated for 30 mins before fluorescence was read at Ex/Em 490/520. Finally, concentration of maleimide residues was determined by interpolation from a standard curve of ethyl maleimide (10 μM–0.01 μM).

### 2.6 Cell labelling with CpG using protein and membrane targeted approaches

CT26 cells were suspended in PBS and incubated with 5 μg of either unmodified Cy5-CpG, Cy5-CpG-Mal or Cy5-CpG-Chol. Cells were incubated at 4 °C for 30 mins before being washed with PBS 3 times. Cells were then acquired on a FACs Calibur flow cytometer before data was analysed using Flowjo software. Fluorescence signal (MFI) above the no treatment control was considered to represent positive association of CpG. An increase in MFI above that obtained for Cy5-CpG indicated an improvement in cell association.

### 2.7 Labelling and isolation of apoptotic cells and sub cellular apoptotic bodies in cell culture

CT26 cells were cultured in a 12-well plate at 1 × 10^5^ cell per ml. Cells were either left unpulsed or pulsed with Dox, Cy5-CpG-Chol or a combination for 24 h. After the elapsed time, apoptotic bodies and cells were isolated in accordance with the centrifugation method used by [20]. In brief, cell supernatant was collected by aspiration and adherent cells were lifted by trypsinisation. The sample was centrifuged at 300 ×g for 15 mins, the resulting pellet was considered the ‘cellular’ fragment, the supernatant was removed and centrifuged at 3000 ×g for 20mins. The pellet of this step was considered to be the ‘cell debris’ containing the sub cellular apoptotic bodies. The two fractions were resuspended in PBS buffer and fractions were assessed for both the presence of Cy5-CpG-Chol and Dox using Flowjo software and presented as contour plots. Annexin V staining of the sub cellular fragment was carried out using an Annexin V kit according to manufacturer's instructions (Biolegend). Gates are drawn on based on unpulsed and/or unstained control, cells whose MFI exceeded the threshold of the gate were considered to be positive for the analysed marker.

### 2.8 Uptake of CpG labelled sub cellular apoptotic bodies by macrophages

The cell debris obtained from the above step was collected from 3 wells and was added to a single well of confluent J774 macrophages in a 12-well plate. Uptake was assessed after by flow cytometry 24 h of culture, cells were first gated on FSC/SSC profile before Cy5-CpG-Chol or Dox was analysed on FL4 and FL2 respectively. Relative uptake was compared by assessing the increase in MFI over unpulsed cells.

### 2.9 Assessment of CpG-Chol tumour localisation by in vivo imaging

Balb/c mice (*n* = 4) were implanted with 1 × 10^6^ CT26 cells subcutaneously on to the right flank. On day 7, once the tumour volume had reached ca.0.5 mm^3^ mice were anesthetised with isoflurane and 7 μg Alexa647-CpG constructs, either with or without Chol, per mouse in 50 μl volume were injected intratumorally. The florescence was tracked using an IVIS Lumina III imaging suite (Perkin Elmer) with excitation/emission set at 620/670 nm. Mice were imaged at the time of injection (0 h), 4 h subsequently and then daily until signal could no longer be detected. Data was analysed using Living Image® 4.3.1 Service Pack 2 software (Perkin-Elmer, UK), automatic ROIs were drawn based on a minimal threshold of 3. In each image a negative non injected mouse was included as a background control. The experiment was terminated when florescence was equal or below background.

### 2.10 *In vivo* co-localization of CpG and Dox

CT26 cells (1 × 10^6^ per tumour) were bilaterally implanted into the rear flank of BALB/c mice. At day 7 tumours were injected i.t. with Dox (20 μg/tumour/mouse) and Alexa647-CpG-Chol (25 μg/tumour/mouse) in 5% dextrose. In each case the right tumour was injected while the left was kept untreated as a negative control. At 24 h post injection, tumours and tumour draining lymph node (TDLN), considered to be the inguinal lymph node on the tumour bearing flank, were excised and cells were dissociated by physical maceration through cell strainer. Tumour derived cells were then washed and acquired on a FACS Calibur flow cytometry. To measure Dox its intrinsic fluorescence was used. For data analysis cells were first gated on FSC/SSC, before being further analysed. Data is therefore expressed as Dox/Alexa647-CpG-Chol as a percentage of high FSC/SSC, the double positive cells (Dox+, Alexa647-CpG-Chol+) indicated cellular co-localisation. Dox and Alexa647-CpG-Chol background was set using the non-treated tumour as a control. For TDLN cells were stained with anti-mouse CD11c-FITC to identify dendritic cells/APCs. Cells were gated on CD11c positive cells before Dox/ Alexa647-CpG-Chol was assessed, signal above non-treated TDLN was considered to indicate uptake.

### 2.11 Evaluation of proposed regime in an in vivo CT26 challenge model

BALB/c mice were implanted unilaterally with 1 × 10^6^ CT26 cells subcutaneously and tumours were allowed to establish for 5 days. On day 5 post implantation, mice were injected i.t. with either 5% dextrose, Dox (20 μg/tumour/mouse/dose), CpG-Chol (25 μg/tumour/mouse/dose) or a combination of the above formulated in 5% dextrose. Mice received further two doses at days 7 and 10 post-implantation. Tumour growth was monitored using a digital calliper and tumour volume was calculated as: Tumour volume = 0.52 × W^2^ × L. The experiment was terminated when the control group mice reached their humane end point. At the final time point, tumours and tumour draining lymph nodes were isolated, cells were disassociated from the tissues using physical maceration through a cell strainer. The single cell suspension from tumours and TDLN was then stained with anti-mouse CD4, anti-mouse CD8 and anti-mouse CD69 monoclonal antibodies. Cells were acquired using a FACs Calibur flow cytometer. Precision count beads were also included in the stain to determine absolute cell number in accordance with manufacturer's protocol. For tumour analysis the parameters investigated were tumour mass and lymphocytes (CD4/CD8) per g of tumour. For TDLN, activated lymphocytes as a percentage of total lymphocytes were assed using monoclonal antibody against early activation marker CD69.

### 2.12 Data analysis

All statistical analysis was performed using Graphpad Prism 8 (La Jolla, California, USA). Flow cytometry data was analysed using Flowjo software (Tree Star, Ashland, OR, USA).

## 3 Results

### 3.1 Membrane reactive CpG is superior to protein reactive CpG for labelling of cells in vitro

**Fig. 1:**
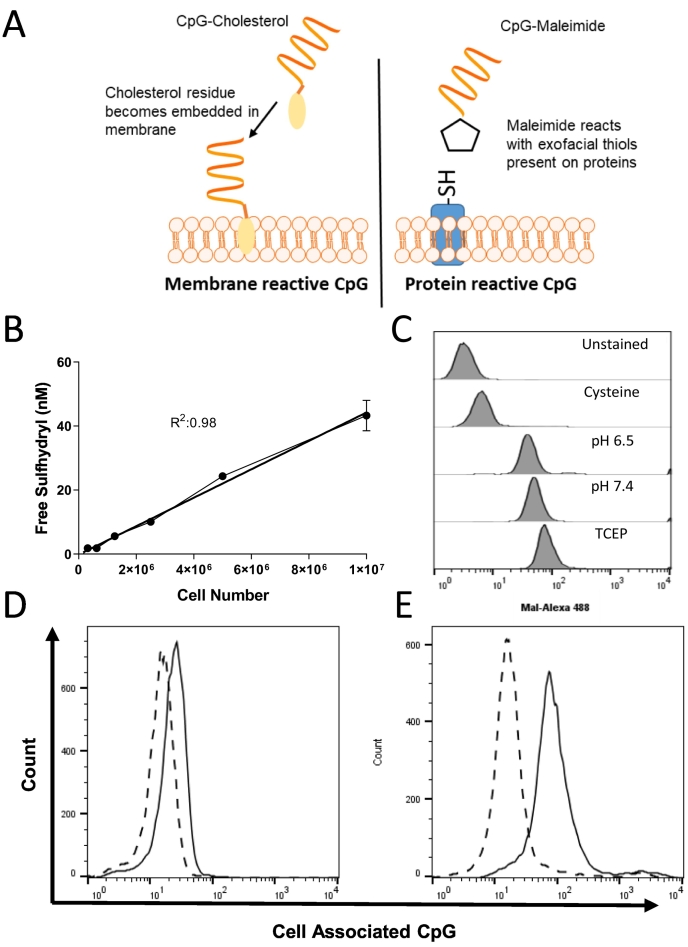
Development of cell surface reactive CpG. Two routes to target CpG to the cell surface were identified: utilising cholesterol or maleimide modified CpG to target cell membrane or protein exofacial thiols, respectively (A). The quantity of free thiol groups on the surface of tumour cell line CT26 was determined using Ellman's reagent against a standard of cysteine (B). Labelling of the cell surface using maleimide chemistries was confirmed using Mal-Alexa488. CT26 cells were incubated with Mal-Alexa488 in the presence of excess cysteine (Cysteine); at pH 6.5 (pH 6.5) or pH 7.4 (pH 7.4); with reducing agent TCEP or left unstained. Cells were acquired using a FACs Calibur flow cytometer (C). Thiol reactive CpG was synthesised from double functionalised CpG (Cy5 CpG NH_2_) after being reacted with SMCC reagent for 1 h and purified using amicon filter column. Membrane reactive double functionalised CpG was synthesised commercially and used without further purification (Cy5 CpG Chol). Each CpG candidate was then mixed with CT26 cells and incubated at room temperature for 30 mins. Cells were washed of excess CpG and analysed using a FACs calibur flow cytometer. Representative flow cytometry histograms for cells labelled with thiol reactive CpG (D, solid line) and membrane reactive CpG (E, solid line) are shown (representative of *n* = 3). In each case a negative control of the corresponding modified CpG was also included (dashed line).

Two approaches to label tumour cell surface were employed, as shown in Fig. 1A: in the first case, CpG was reacted with sulfo-SMCC to generate maleimide functionality; in the second case CpG was conjugated to cholesterol which will insert into the cell membrane. Pursuing the first approach, thiols residues on the cell membrane protein were used to introduce CpG moieties as the maleimide residue is reactive to reduced exofacial thiol residues on proteins. The presence of exofacial thiols was determined using Ellman's reagent. As shown in Fig. 1B, there was a linear relationship between cell number and thiol groups with a strong correlation. To confirm these residues were accessible to partake in the maleimide reaction, maleimide-Alexa488, was mixed with CT26. Alexa488 is a hydrophilic dye and as such is membrane impermeable thus only thiol residues on the cell surface will be labelled. As shown in Fig. 1C, cells showed a high degree of labelling compared to control. The inclusion of excess free cysteine in this labelling reaction completely ablated this effect suggesting thiol specificity. Performing the reaction in the presence of media pH 6.5, mimicking tumour pH, had no effect on the labelling efficiency. The inclusion of a reducing reagent TCEP, to reduce disulphide bonds, marginally improved labelling efficiency.

Having established the presence of reduced thiols on the cell surface, we next sought to determine whether these thiols were accessible *in vivo*. As shown in Suppl. Fig. 2, a single injection of Mal-Alexa488 intratumorally resulted in specific cell labelling above the negative control (Azide-Alexa488) suggesting the thiol groups are a suitable *in vivo* target. Though, it should be noted, the phenotype of the labelled cells was not determined.

**Fig. 2:**
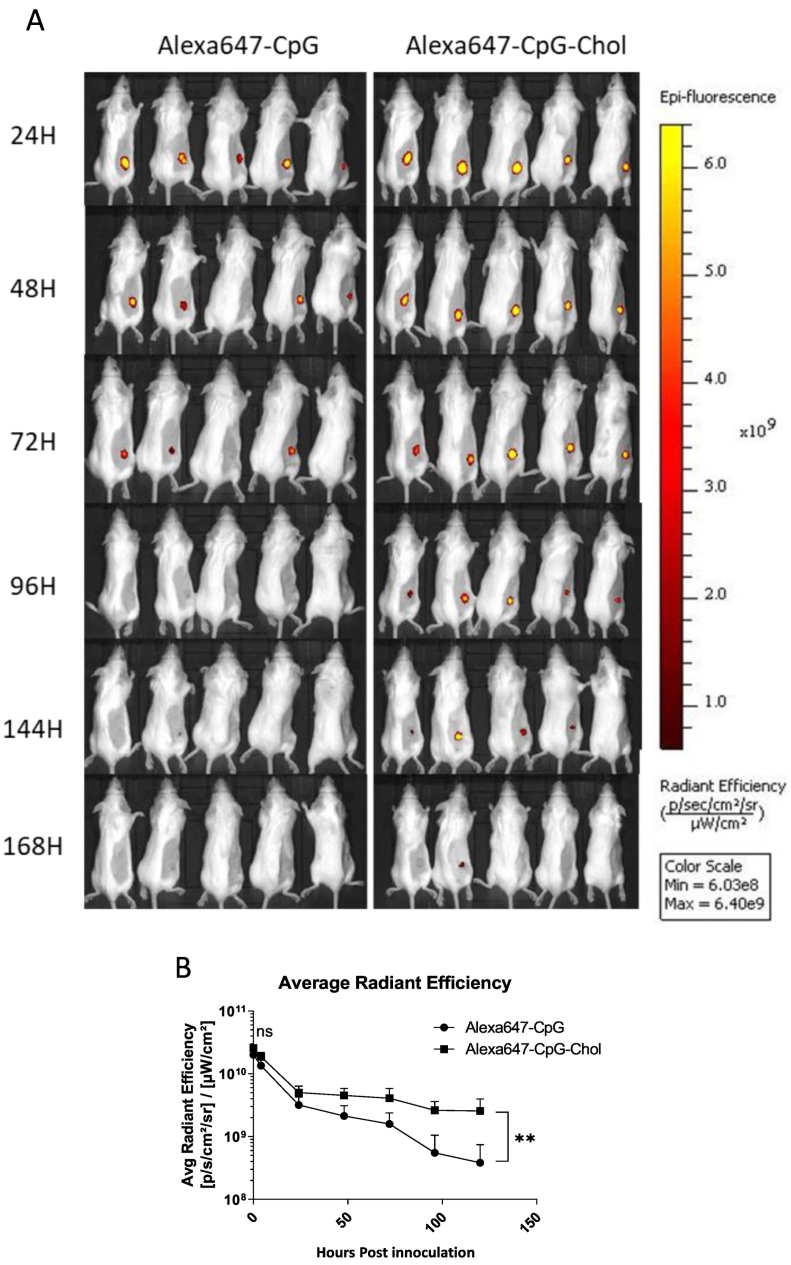
Cholesterol modified CpG is retained at injection site significantly longer than unmodified CpG. Balb/c (*n* = 5) were implanted with CT26 cells (1 × 10^6 per mouse) subcutaneously. After 10 days when tumours reached approximately 0.5mm^3^ volume mice were injected i.t. with 7μg of either Alexa647-CpG or Alexa647-CpG-Chol. At the appropriate time interval mice were imaged using an IVIS III Lumina imaging system, the fluorescence was tracked following injection (A). Data shows the mean of the average radiant efficiency per group (B), error bars correspond to the standard deviation. Data was analysed using *t*-test followed by Mann Whitney post test *p < .05 ns non significant Graphpad 5.

In the next stage, the protein and membrane targeting Cy5-CpG were compared in terms of cell association. Amine modified Cy5-CpG was reacted with SMCC to generate maleimide functionality, this was confirmed using maleimide detection kit (suppl. 1). When reacted with CT26, it was observed that the presence of maleimide functionality on CpG, unlike Mal-Alexa488, only slightly increases cell association above background. In contrast CpG-Chol improves CpG-cell association by nearly 10-fold (Fig. 1D-E). In both cases there was a high degree of background association, greater than the background using Mal-Alexa488, was observed which may be why the results differ. As a result of these data CpG-Chol was selected as our most viable candidate to proceed further.

### 3.2 Alexa647-CpG-Chol persists at injection site significantly longer than unmodified CpG

We hypothesised that the Alexa647-CpG-Chol construct would be retained for an extended period at the tumour site due to increased cell labelling. This prolonged retention would increase the window in which both Dox and CpG are present in the tumour simultaneously thus theoretically permitting the formation of a greater number of apoptotic fragments-CpG complexes. Alexa647 was used in place of Cy5 as we speculated, due to the hydrophobic nature of Cy5, there may be more nonspecific cellular interaction. The persistence of the Alexa647-CpG-Chol construct was compared to unmodified Alexa647-CpG *in vivo* (Fig. 2). Following intratumoral injection there was a rapid decline in fluorescence, with both Alexa647-CpG constructs losing nearly 90% of the signal between 0 and 24 h suggesting lymphatic drainage. The signal intensity then steadily decreased between 24 and 120 h with Alexa647-CpG-Chol persisting for a significantly longer time than unmodified CpG. At the end of the time course there was approximately double the Alexa647-CpG-Chol persisting at tumour site when compared to unmodified Alexa647-CpG. From 72 to 96 h post injection 40% (2 of 5) mice in the unmodified Alexa647-CpG group had no detectable fluorescence compared with 0% in Alexa647-CpG-Chol group. This is further evidence that Alexa647-CpG-Chol cell labelling is significantly better than unmodified Alexa647-CpG.

### 3.3 Alexa647-CpG-Chol can label both apoptotic cells and sub cellular apoptotic bodies *in vitro*

**Fig. 3:**
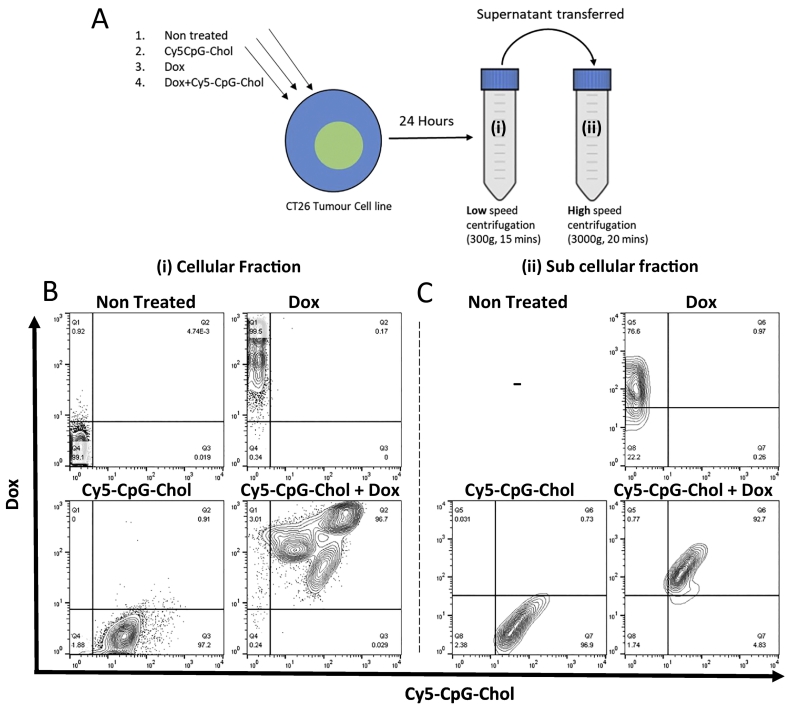
CpG-Chol and Dox are present in treated cells and sub cellular apoptotic bodies. CT26 Cells were treated with either Dox, Cy5-CpG-Chol, Dox + Cy5-CpG-Chol or left untreated as described following 24 h incubation. Intact cells were collected by low speed centrifugation (300 ×g 15mins) and apoptotic bodies were collected by high speed centrifugation (3000 ×g 20mins) and washed 3 times (A). Fractions were acquired on a FACs Calibur flow cytometer with previously optimised settings. Gates were drawn based on unstained controls. Analysis was performed using Flowjo software. The intrinsic Dox fluorescence plotted against Cy5-CpG-Chol signal is shown in representative flow cytometry plots for cellular (B) and sub cellular (C) fraction.

Having established the ability of Alexa647-CpG-Chol to associate with cells *in vitro*, and longer persistence *in vivo*, the ability of CpG to become associated with Dox treated cells and sub cellular fraction following treatment with an ICD inducing drug was determined. As shown in Fig. 3A, CT26 cells were either left unpulsed or pulsed with Alexa647-CpG, with or without Dox. After 24 h incubation the cellular and sub cellular fractions were separated from each other using differential centrifugation. The sub cellular fraction was tested to confirm it contained fragments arising as a result of apoptosis by staining with annexin V (Suppl. Fig. 4). Both fractions were then analysed for the presence of Dox and Alexa647-CpG-Chol (Fig. 3B-C). As expected, untreated cells exhibited minimal Dox or CpG fluorescence signal, furthermore, we were unable to obtain a sufficient sub cellular fraction to analyse. In contrast, nearly all Dox treated cells (99.5%) took up the drug, reflective of this, 76.6% of sub cellular fragments became positive for Dox. Similarly, individual treatment with Alexa647-CpG-Chol caused 97.2% of cells to give a positive signal. Within the sub-cellular fraction, 96.9% of fragments detected were positive for Alexa647-CpG-Chol. When Alexa647-CpG-Chol and Dox were combined >95% cells were positive for Alexa647-CpG-Chol and Dox. The sub cellular fragment stained highly with both Dox and Alexa647-CpG-Chol with 92.7% of the events being double positive. Taken together, these data suggest that following Alexa647-CpG-Chol + Dox treatment, both CpG and Dox become incorporated into the cell, and that this incorporation is maintained following cell death.

**Fig. 4:**
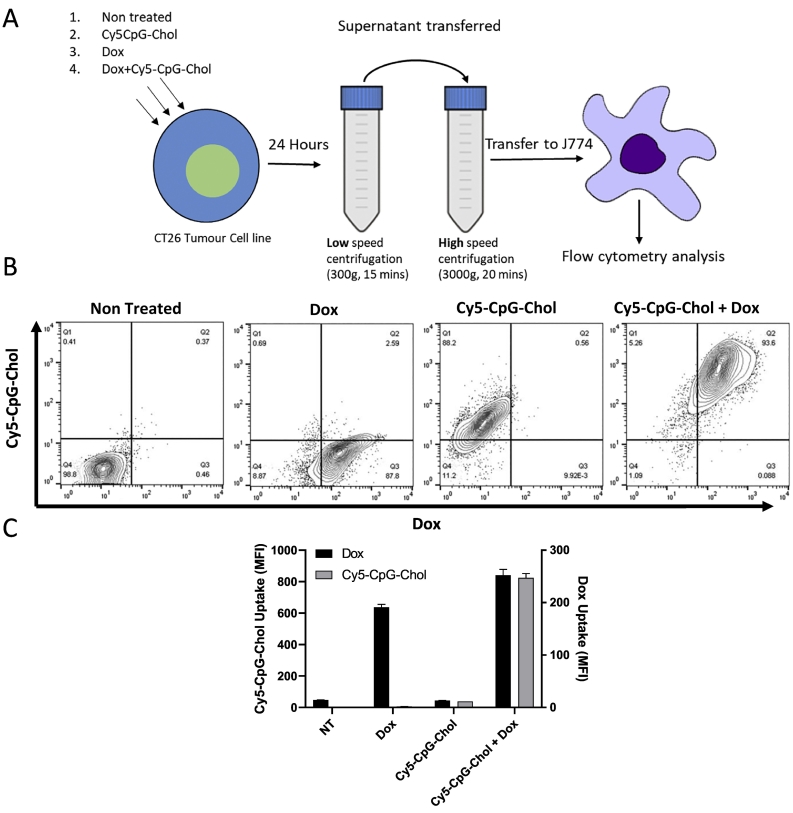
CpG-Chol labelled apoptotic bodies are taken up by J774 macrophages *in vitro.* CT26 Cells were treated with either Dox, Cy5-CpG-Chol, Dox + Cy5-CpG-Chol or left untreated. Following 24-h incubation intact cells were removed by low speed centrifugation (300 ×g 15mins) and apoptotic bodies were collected by high speed centrifugation (3000 ×g 20mins) and washed 3 times. The whole apoptotic body fraction from a single well was transferred to a single well of J774 macrophages and co incubated for 30 mins. Following the incubation period, J774 cells were washed 3 times and uptake of apoptotic bodies was assessed by flow cytometry (A), the intrinsic fluorescence of Dox is plotted against Cy5-CpG-Chol (B), the MFI values obtained for both Dox and Cy5-CpG-Chol is plotted in (C).

### 3.4 Sub cellular apoptotic bodies can transfer both CpG and Dox to J774 macrophages *in vitro*

In the current model it is hypothesised that apoptotic bodies produced as a result of Alexa647-CpG-Chol + Dox treatment will be taken up by cells either locally, within the tumour, or more distally, within the lymph node, acting as a source of antigen-adjuvant complex. To model transfer of CpG apoptotic body complex to phagocytic cells, the uptake of the sub cellular fragments by macrophages was tested (Fig. 4A). J774 macrophages were pulsed with the sub cellular fraction generated from CT26 cells that had either been left untreated, treated with Dox, Alexa647-CpG-Chol or Alexa647-CpG-Chol + Dox. Fig. 4B shows the Alexa647-CpG-Chol plotted against intrinsic fluorescence of Dox. Sub cellular fraction obtained from non-treated cells shows minimal background, the individual monotreatments of Dox and Alexa647-CpG-Chol caused J774 to become positive for the corresponding dye suggesting transfer of either CpG or Dox is possible through the sub cellular fraction. The double treatment resulted in J774 cells becoming highly positive for both CpG and Dox. This data is shown graphically in Fig. 4C.

### 3.5 Dox and Alexa647-CpG-Chol are co-localised in cancer cells vivo and Alexa647-CpG-Chol can be detected in CD11c + cells in TDLN

**Fig. 5:**
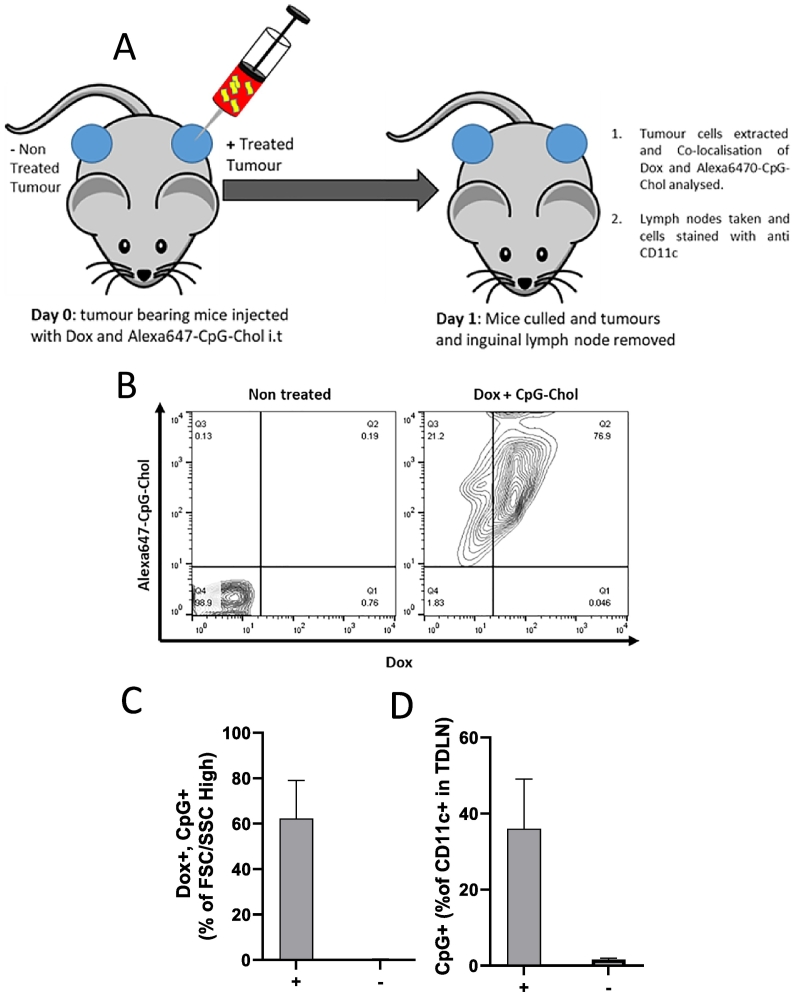
CpG and Dox are co localised in tumour cells *in vivo* following i.t injection. CT26 cells were bilaterally implanted into BALB/cmice(n=5pergroup). At day 7 post implantation mice were injected intratumorally with Dox (20ug/mouse/dose) plus Chol-CpG-Alexa647 (25ug/mouse/dose) into the right tumour (+), the left tumour served as negative control and was left uninjected (-) (**A**). At 24h post injection the tumour and tumour draining lymphnode were removed and cells were extracted by physical maceration. Cells extracted from the tumour were washed and acquired on a FACs Calibur flow cytometer. Cells were first gated based on FSC/SSC, with unstained cells serving as the negative control, before CpG and Dox fluorescence were analysed. A representative flow cytometry plot is shown in (**B**), the data is represented graphically in (**C**). TDLN cells were stained with antimouse CD11c-FITC to identify DC population before being acquired as above. Data was analysed by first gating on CD11c positive cells before CpG uptake was assessed, Dox could not be detected above background in TDLN (**B**).

To determine whether the process described *in vitro* can occur *in vivo*, tumour bearing mice were injected i.t. with both Alexa647-CpG-Chol and Dox. Tumours were excised and cells extracted were analysed for the presence of Alexa647-CpG-Chol and Dox. As shown in Fig. 5A-B, in tumours receiving combination treatment, 60% of events were also positive for both Alexa647-CpG-Chol and Dox indicating that the two agents are co-localised to the same cell. There is minimal background fluorescence from the bilateral non injected tumour. This strongly supports the proposed hypothesis and that events observed *in vitro,* as described in the prior sections, are likely to be occurring *in vivo*. It should be noted that in this study propidium iodide could not be used due to spectral overlap with Dox. The cellular co-localisation of Dox and Alexa 647-CpG-Chol strongly suggests the *in vivo* conditions can recapitulate *in vitro* setting. Within the TDLN it was observed that nearly 40% of CD11c + cells were positive for Alexa647-CpG-Chol in the treated tumour. Dox could not be detected within the TDLN possibly due to the weak fluorescence of Dox and dilution of signal.

### 3.6 *In vivo* assessment of proposed regime in tumour challenge model

**Fig. 6:**
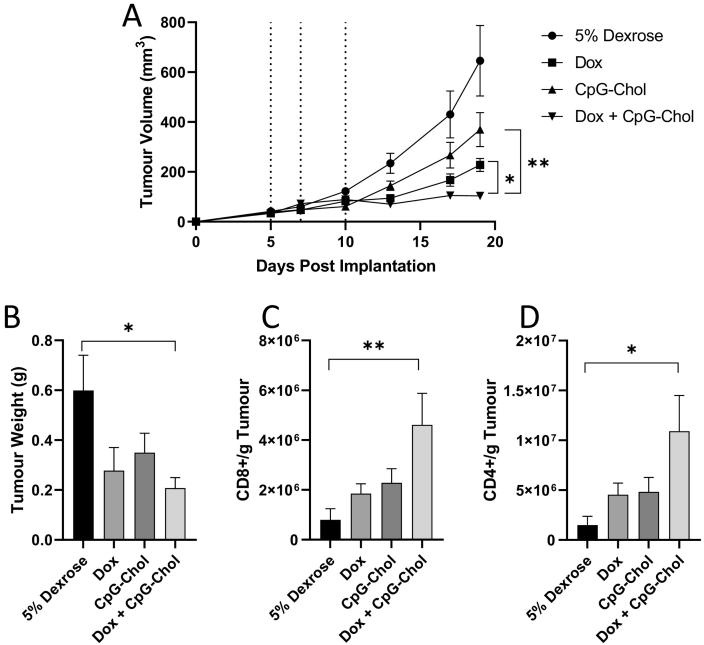
Effect of mono or combined therapies on tumour growth and cell infiltrates *in vivo*. CT26 cells were implanted into the lower flank of BALB/c mice (*n* = 9 per group). At day 5 post implantation, when tumours had become palpable, mice were injected intra tumorally with either: 5% dextrose, Dox (20 μg/mouse/dose), CpG-Chol (25 μg/mouse/dose) or CpG-Chol + Dox (25 μg and 20 μg/mouse/dose). Tumours were measured and a second, and third dose was administered at days 7 and 10 respectively (dashed lines) (A). Data plotted represents mean and SEM. *p < .05, **p < .005, two-way ANOVA followed by Tukey's pos*t*-test. At the terminal end point tumours were extracted and weighed (B). Cells were isolated from tumours using physical dissociation and stained with anti-mouse CD4 and anti-mouse CD8 conjugated monoclonal antibodies. Cells were enumerated using precision count beads and presented as number of CD8+ (C) or CD4+ (D) cells per mg of tumour. Data plotted represents mean and SEM. *p < .05, **p < .005 Student's *t*-test.

To establish the effect of this regime *in vivo*, mice bearing CT26 tumours were injected intratumorally with 3 doses of either 5% dextrose, Dox, CpG-Chol or CpG-Chol+Dox at days 5, 7 and 10 post tumour implantation. Having observed, increased cellular association and prolonged persistence of CpG-Chol, in accordance with other studies on lipid modified CpG, we did not include the unmodified version of CpG in this study [21]. Tumour size was monitored and is reported in Fig. 6. As shown in Fig. 6A, 5% dextrose treated mice reached their humane end point at day 19 following implantation. Treating the tumours with CpG alone resulted in a non-significant reduction of tumour size of approximately 30% volume compared to buffer alone at the terminal time point. We observed no significant indication of toxicity in any of the groups (data not shown). Predictably, Dox was highly effective in delaying tumour growth with tumour volumes reaching, on average, 227 mm^3^ compared to 645 mm^3^ in the 5% dextrose group on day 19 (post cull). The effect of Dox was significantly enhanced by the inclusion of CpG-Chol in the regime with tumours volumes in this group reaching only 103mm^3^, roughly half the volume of Dox alone group. At the terminal end point the trend in tumour weight followed the trend in volume, however, due to variations between animals, significance was only obtained between the 5% Dextrose and Dox + CpG groups (Fig. 6B). This data suggests that there is either a synergistic or a cumulative effect of Dox and CpG on tumour growth.

To establish whether this effect was due to immune modulation, cell subsets within the tumour were analysed. Interestingly, Dox + CpG strongly enhanced cell infiltration resulting in nearly double the cell density of CD8+ (4 × 10^6^ vs 2.2 × 10^6^ CpG Chol vs 1.8 × 10^6^ Dox) (Fig. 6C) and CD4+ (1 × 10^7^ vs 4.8 × 10^6^ CpG-Chol vs 4.5 × 10^6^ Dox) (Fig. 6D) cells compared to either of the individual treatments. The ratio of CD4+ to CD8+ was maintained as was the ratio of FOXP3+ CD4+ cells (data not shown) suggesting expansion of the compartment rather than being subset specific. It should also be noted the functionality of these T cells was not analysed. Surprisingly, mono-treatment with CpG or Dox were equivalent in their ability to elevate CD8+/CD4+ populations.

**Fig. 7:**
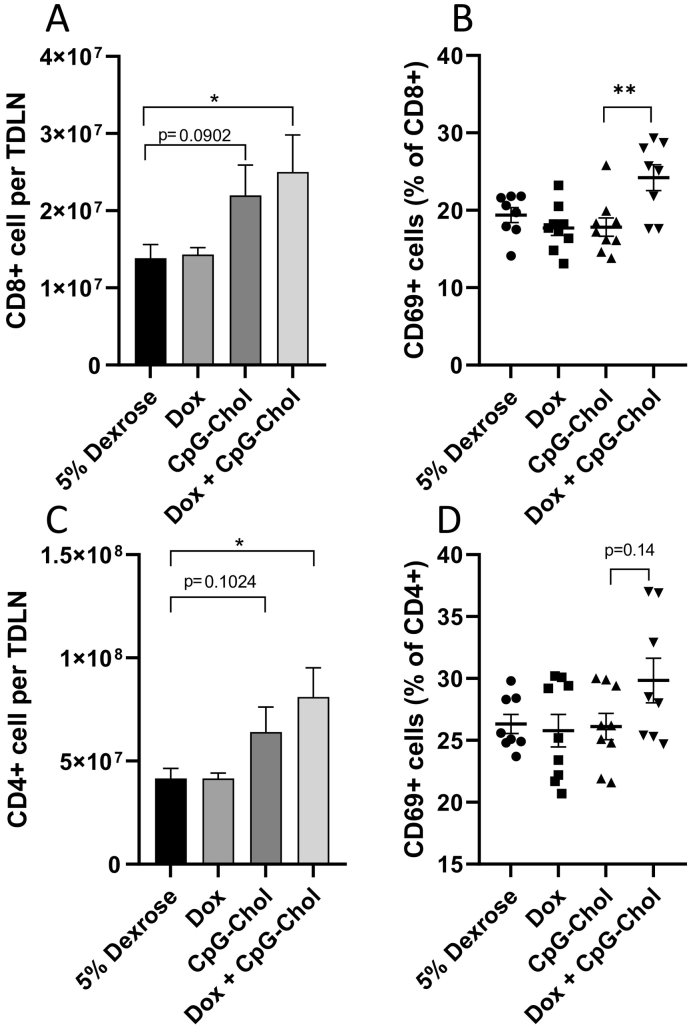
Effect of mono or combined therapies on tumour draining lymph node (TDLN) cell subsets. TDLN were extracted from CT26 tumour bearing BALB/c mice following three metronomic treatments of either 5% dextrose, Dox (20μg/mouse/dose), CpG-Chol (25μg/mouse/dose) or CpG-Chol + Dox (25μg and 20μg/mouse/dose) on days 5, 7 and 10 post implantation (n=9). Cells were isolated and stained with anti mouse CD4, CD8 and CD69 fluorophore conjugated monoclonal antibodies. Total CD8 (**A**) and CD4 (**C)** cell counts per lymph node were obtained using precision count beads. To assess CD69 expression in the relevant populations, cells were gated on either CD8+ (**B**) or CD4+ (**D**) prior to analysis. Data is presented as CD69+ as a percentage of parent population. **p<0.005 Student’s T Test.

The increased immune stimulation observed in the tumour with CpG + Dox was further manifested in the tumour draining lymph node (TDLN). As shown in Fig. 7 A&C, there were elevated numbers of both CD4+ and CD8+ cells in the CpG + Dox group with was significantly different from the 5% dextrose control but not significantly different from CpG alone which in turn did not obtain statistical difference from the buffer control. The activation state of the lymphatic cells was assessed by monitoring the expression of the early activation marker CD69. As a percentage of the CD8+ compartment, there were significantly more CD69+ cells in TDLN extracted from mice receiving Dox + CpG (average: 24%) compared to any other group (average across all groups: 18%) post cull suggesting cells have received stimulation (Fig. 7B). While this trend was maintained within the CD4+ population, significance was not obtained due to intra group variation (Fig. 7B). As there was no elevation in CD69+ CD8+/CD4+ cells in any group other than Dox + CpG-Chol this strongly suggests that this is a synergistic, rather than a cumulative effect, between CpG-Chol and Dox in the TDLN.

## 4 Discussion

This manuscript describes the use of cell membrane reactive immune adjuvant to generate an *in situ* ‘apoptotic cell adjuvant complex’. Previously, groups have used maleimide reactivity to conjugate CpG containing lipid particles to apoptotic cells generated *in vitro*, and to T cells for enhancement of adoptive T cell therapy [22]. In the current study it was observed that the tumour cell utilised (CT26) possess reactive surface thiols. This was also the case with other cell lines we tested (4 T1 and B16F10 (Suppl. Fig. 3)) suggesting the presence of exofacial thiols is somewhat conserved amongst laboratory cancer cell lines and may represent a potential future target for *in situ* vaccination. Indeed, it has been previously suggested that maleimide targeting of thiol groups may improve uptake of biomolecules [23]. We, however, observed minimal enhancement of cell association of maleimide CpG compared to free CpG. This may be an artefact of the *in vitro* system, or because of hydrolysis of maleimide groups during conjugation or in the wash steps of purification, as maleimide modified Alexa488 greatly improved association. For *in vitro* and *in vivo* studies, based on cell association studies, we chose to use membrane reactive CpG-Chol. Conjugation to cholesterol has been previously been investigated as a means of targeting CpG to the cell membrane and was shown to be effective; however, constructs with diacyl fatty acids chains were shown to be superior [21]. Indeed diacyl conjugates have been used in a number of studies looking at post insertion into cell membranes [24]. For this preliminary investigation Chol was chosen based on ease of synthesis and commercial availability. The CpG construct was modified with Chol at the 3′ terminal as it has been reported that modification at the 5′ terminal impairs function, though this is not consistent in the literature [25,26].

We opted to use a CpG ODN 1668 construct which has previously been used by our group and has been used in intratumoral models [19,27]. CpG ODN 1668 has been shown to be highly effective at activating and priming T cells in the TDLN after following DC migration from the tumour, a feature which we hypothesised may be advantageous in our model [27,28]. Consistent with our observations, previous reports using CpG 1668 intratumorally did not show any elevation of CD4+/CD8+ intratumoral cell numbers when CpG single treatment was used [27]. Likewise, when used in isolation, CpG 1668 did not increase CD69 expression on splenocytes, as we observed in the TDLN [29]. In the current study we observed both elevated cell numbers intratumorally and increased activation of CD4+/CD8+ cells only in the CpG-Chol, Dox combination group suggesting potential synergy between the two. Within the literature the use of the CpG ODN 1826 construct is more common and has shown to be highly effective against CT26 [[30], [31], [32], [33]].

In our model, the combination of ICD inducing drug (Dox) and surface reactive adjuvant (CpG-Chol) resulted in the most promising tumour growth suppression. Dox and CpG have previously been formulated together in a microparticulate system and as a plasmid DNA-Drug complex [34,35]. In both the prior studies complex immunological characterisation was not performed; however, when microparticles were used, local treatment of tumours could resolve distal tumours suggesting systemic immunity. Our candidate CpG-Chol formulated with Dox, injected intratumorally, potentially circumvents many regulatory/biomanufacturing translational pitfalls of more complex particulate systems while maintaining efficacy.

Although we have strong data to suggest apoptotic body-adjuvant complexes can be formed *in vitro* and that Dox and CpG are co-localised *in vivo*, we have yet to be able to isolate the complex *ex vivo.* Nevertheless, to our knowledge, this is the first *in vitro* description of co localisation of adjuvant and drug within subcellular apoptotic bodies, the existence of these complexes *in vivo* may have profound influence on potential drug bystander effect in addition to drug adjuvant synergy. We also speculate that our approach may be advantageous over those using particulate/complex co-formulation as it proposes the apoptotic fragment and adjuvant are co-localised and delivered to the same cell which been shown to be improve immunogenicity in a number of models [29,36]. As a next step our regime could be tested with checkpoint blocking antibodies such as anti PD-L1, CTL4 or OX40 antibodies which have shown synergy with CpG [37]. To obtain greater specificity for apoptotic fragments CpG could be bound to molecules such as annexin V or ApoPep-1 reactive against phosphatidylserine and histone H1 respectively, both associated with apoptosis [38].

## 5 Conclusions

This manuscript describes the development of a cell surface reactive adjuvant for labelling cells undergoing immunogenic cell death, we have gathered good evidence to suggest that adjuvant apoptotic body complexes exist *in vitro*. Furthermore, that the circumstances in which this complex is generated can replicated *in vivo* through intratumoral injection. Therapy using the described approach leads to delayed tumour growth, increases cell infiltration and activation.

## Author contributions

The manuscript was written through contributions of all authors. All authors have given approval to the final version of the manuscript.

## Declaration of Competing Interest

The authors have declared that no competing interest exists.

## Appendix A Supplementary data

Supplementary material Image 1
