# Supplementary material for: Evaluation of cell surface reactive immuno-adjuvant in combination with immunogenic cell death inducing drug for in situ chemo-immunotherapy
Source: J Control Release. 2020 Jun 10;322:519–29. doi: 10.1016/j.jconrel.2020.03.029 (PMC7262586; doi:10.1016/j.jconrel.2020.03.029)
Supplement: Supplementary file 1 — Supplementary material [file mmc1.pdf]

Supplementary Information

Evaluation of cell surface reactive immuno-adjuvant in combination with immunogenic cell death inducing drug for in situ chemo-immunotherapy

Adam A Walters[a,*], Julie Tzu-Wen Wang[a] and Khuloud T. Al-Jamal[a,*]

[a] Institute of Pharmaceutical Science, Faculty of Life Sciences & Medicine, King's College London, Franklin-Wilkins Building, 150 Stamford Street, London SE1 9NH, United Kingdom

[*]Corresponding authors

Prof. Khuloud T. Al-Jamal

Email address: khuloud.al-jamal@kcl.ac.uk

Dr Adam Walters

Email address: adam.walters@kcl.ac.uk

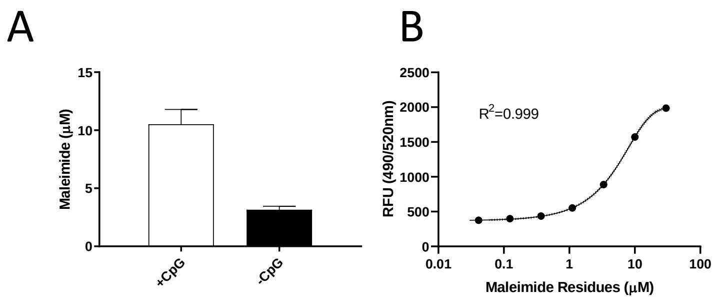

Suppl. Fig 1. SMCC can be used to functionalise CpG-NH_2 with maleimide residues CpG-NH_2 (10µM) was incubated with SMCC as a previously determined optimal ratio for 1 hour. CpG was purified and washed using an amicon 3k MWCO spin column. The presence of maleimide was determined using a maleimide detection kit (**A**). A standard of SMCC was used to quantify residues (**B**). As a negative control, a 'mock' conjugation, which lacked CpG, was performed (filled bar). Data represents mean and SD.

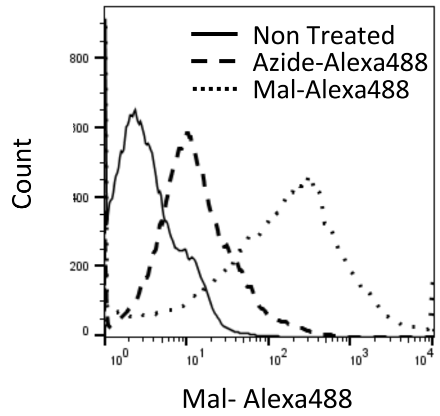

Suppl. Fig 2. *In vivo* accessibility of reactive thiol residues. The availability of thiols *in vivo* was assessed using Mal Alexa 488. BALB/c mice (n=3) bearing CT26 tumours were injected i.t. with either Mal-Alexa488 (dotted line), Azide-Alexa488 (dashed line) to act as a background control or left untreated (solid line) at day 10 post implantation. Tumours were removed 24 hours subsequently and cells extracted using physical dissociation. Fluorescence was detected using FACs Calibur flow cytometer.

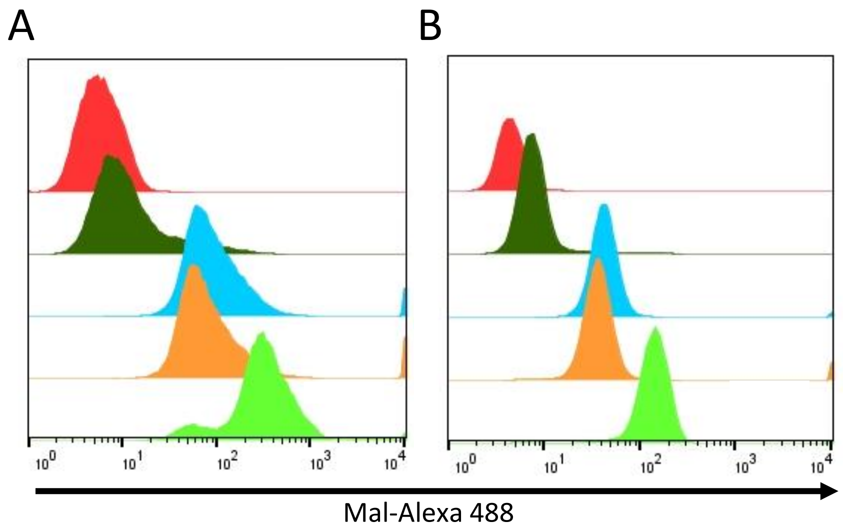

Suppl. Fig 3. Identification of free exofacial thiols on common laboratory cancer cell lines. Labelling of the cell surface of laboratory cancer cell lines was tested using Mal-Alexa488. Both 4T1 breast cancer (**A**) and B16F10 melanoma (**B**) cells were incubated with Mal-Alexa488 in the presence of excess cysteine (Dark Green); in at pH6.5 (Blue) or pH7.4 (Orange); with reducing agent TCEP (Light Green) or left unstained (Red). As described for CT26. Post staining, cells were acquired using a FACs Calibur flow cytometer.

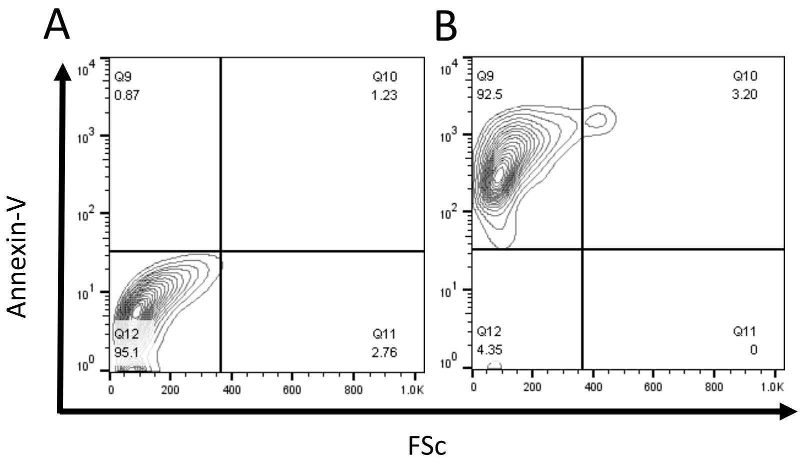

Suppl. Fig 4. Sub cellular isolate stains positively with annexin V. CT26 Cells were treated with either Dox for 24 hour incubation subcellular fraction was collected with high speed centrifugation (3000xg 20mins) and washed 3 times. The collected fraction was either stained with left unstained (**A**) or stained with annexin V-FITC conjugate (**B**). The sample was then acquired on a FACs Calibur flow cytometer.
